# The Life Skills of Older Americans: Association with Economic, Psychological, Social, and Health Outcomes

**DOI:** 10.1038/s41598-018-27909-w

**Published:** 2018-07-05

**Authors:** Andrew Steptoe, Sarah E. Jackson

**Affiliations:** Department of Behavioural Science and Health, University College London, London, UK

## Abstract

Studies of children and adolescents indicate that success in life is determined in part by attributes such as conscientiousness, emotional stability and sense of control, independently of childhood socioeconomic status and cognitive ability. Less is known about the role of these characteristics at older ages. This study investigated the relationship of five life skills – conscientiousness, emotional stability, persistence, optimism and sense of control – with a range of outcomes in 8,843 participants (mean age 72.57 years) in the Health and Retirement Study, a representative study of older Americans. More life skills were associated with greater wealth and income, better emotional wellbeing, stronger social relationships, less loneliness, better health, fewer chronic illnesses and impaired activities of daily living, better mobility and less obesity, after controlling for childhood socioeconomic status and current cognitive ability. Longitudinally, more life skills predicted emotional wellbeing, less loneliness and more prosocial behavior, better health and mobility over a 4 year period. Associations were independent of gender, ethnicity, family background, education and cognitive ability. The number of attributes was important rather than any single life skill. Life skills continue to matter at advanced ages, and fostering these characteristics in older adults may pay dividends in terms of later life health and wellbeing.

## Introduction

Life skills comprise a set of personal, social and emotional characteristics and capabilities including conscientiousness, emotional stability, optimism, persistence and determination, sense of control and social skill that are thought to impact success in life^1,2^. These factors are often described as ‘non-cognitive’ to distinguish them from intellectual ability, and are an emerging focus of educational and developmental programs^3,4^. There is moderate consensus on what features are most important among these attributes. A report for the Education Endowment Fund in the UK listed self-efficacy, goal orientation, intrinsic motivation, perseverance, grit, self-control, social competencies such as leadership and social skills, and creativity, and their relationship with academic achievement, emotional health and prosocial behavior among young people^1^. Putnam argued that grit, social sensitivity, optimism, self-control, conscientiousness, and emotional stability are important for life success^5^. Other authorities have highlighted control, self-esteem and determination^2,6^.

It is notable that several of these factors are associated with favorable outcomes in adult life as well. For example, conscientiousness, optimism, sense of control and emotional stability have been shown in longitudinal population studies to predict greater longevity^7–11^. These factors also predict reduced incidence of mental ill-health and chronic physical diseases such as diabetes^12–14^. Persistence, emotional stability, conscientiousness and optimism are associated with life satisfaction, economic success, more stable social relationships and prosocial behavior^15–17^. The same factors are also associated with favorable biomarkers^18–20^. However, most studies have investigated these factors separately, and there is limited evidence about whether the accumulation of life skills is important in later years.

We therefore carried out an analysis of the English Longitudinal Study of Ageing (ELSA) to study the relationship between number of life skills and a range of outcomes in men and women aged 50 and older^21^. Measures of five life skills were available in that dataset – conscientiousness, persistence, emotional stability, optimism and sense of control. We found that the number of life skills possessed by participants was associated with greater wealth and income, greater subjective wellbeing, less depression, less social isolation and loneliness, more prosocial behavior, better self-rated health, fewer chronic diseases and impaired activities of daily living, faster walking speed, and healthier biomarker profiles^21^. No single life skill appeared to be responsible for these associations, since they were maintained after each attribute was removed in turn from the cumulative index. The present study sought to replicate and extend these findings with a representative sample of older Americans, examining both cross-sectional and longitudinal relationships between the number of life skills and a range of economic, psychological, social and health outcomes. We measured the same set of life skills as in ELSA for comparability, and were able to add additional outcomes. The analyses took account of age, sex, ethnicity, family socioeconomic background, education, and cognitive ability, so as to investigate the impact of life skills independently of early life circumstances and current cognition.

## Methods

### Study population

Data were analyzed from the Health and Retirement Study (HRS), a longitudinal study of Americans aged 50 years and older^22^. Assessments are made every two years, with some measures being administered to 50% of the sample on one wave and to the remaining 50% two years later. Baseline measures were obtained by combining waves in 2008 and 2010, while longitudinal analyses primarily involved assessments in 2014. A total of 8,843 participants with data on all five life skills and covariates were included in the analyses.

### Measurement of life skills

The five life skills were measured by combining ratings obtained in 2008 and 2010. Conscientiousness and emotional stability were measured using the Midlife Development Inventory (MIDI) Personality Scales^23^. Participants rated the extent to which various items described them on a 4-point scale ranging from *not at all* to *a lot*. Items contributing to conscientiousness include ‘responsible’ and ‘thorough’, while the emotional stability scale included ‘nervous’ and ‘calm’. Persistence was assessed with a single item concerning the extent to which participants had felt ‘determined’ over the past 30 days (responses ranged from *not at all* to *very much*). Optimism was measured with a shortened version of the Life Orientation Test, consisting of three items (‘I’m always optimistic about my future’, ‘In uncertain times, I usually expect the best,’ and ‘Overall, I expect more good things to happen to me than bad’), each of which was rated from 1 = *strongly agree* to 6 = *strongly disagree*^24^. Sense of control was indexed by the single item ‘I have little control over the things that happen to me’ rated from 1 = *strongly disagree* to 6 = *strongly agree*^25^. Respondents were considered to express the life skill at a high level if they were in the top quartile of the distribution for conscientiousness, emotional stability and optimism, if they rated their persistence as *very much*, and responded *strongly disagree* to the control item. The life skills index was computed by adding the number of high level skills, so could range from 0 to 5. Because of limited numbers of respondents with all 5 skills, the 4 and 5 skill categories were collapsed for purposes of analysis.

### Covariates

Age was modeled as a continuous variable. Ethnicity was categorized into White, Black, and Other. Childhood socioeconomic status was indexed in terms of the father’s education, and classified as lower, intermediate, and higher based on years of schooling. The participant’s own educational attainment was categorized into less than high school, high school, some college, and college and higher. Cognitive capacity at baseline was measured by aggregating immediate and delayed recall, and responses to counting, naming, and vocabulary tasks, orientation items, serial 7s performance, and backwards counting, as detailed in RAND HRS data documentation^26^. Marital status was not included as a covariate, since preliminary analyses indicated that it was not associated with number of life skills, and it did not modify the associations of life skills with other outcomes.

### Outcomes

#### Economic measures

Wealth was computed from a detailed assessment of the participant’s economic resources, and included financial, housing and physical wealth but excluded pension wealth. Income was calculated as total weekly net family income from all sources including employment, state benefits, pensions and other assets. Cross-sectional analyses were based on the proportion in each life skill category who were in the highest wealth or income quintile, though comparable results emerged when wealth or income was modeled as continuously distributed variables. In 4-year longitudinal analyses, we analyzed the proportion of people in each life skill category in the highest quintile of wealth or income category in 2014, adjusting for baseline wealth.

#### Psychological measures

Depressive symptoms were measured using the 8-item Center for Epidemiologic Studies Depression Scale (CES-D) with binary response categories, and a score of ≥4 was taken to indicate significant symptomatology, as used in other investigations^27^. Anxiety was assessed using 5 items from the Beck Anxiety Inventory, and was modeled as a continuous score ranging from 1 (low) – 4 (high)^28^. Financial strain was measured with the single item ‘How difficult is it for you/your family to meet monthly payments on your bills?’ There were 5 possible responses (*not at all difficult, not very difficult, somewhat difficult, very difficult*, and *completely difficult*). Endorsement of one of the last three categories was defined as financial strain. Chronic stressors were assessed with ratings of 7 sources of stress (e.g. ongoing housing problems, ongoing physical or emotional problems in spouse or child), rated from 1 = *no, didn’t happen* to 4 = *yes, very upsetting*^29^. Analyses were based on mean ratings. Chronic stressors were not assessed in 2008 or 2014; consequently, baseline analyses were carried out on a reduced sample, and longitudinal analyses on assessments in 2012.

#### Social measures

Social isolation was assessed using an index of the extent of contact with children, other family members, and friends, and participation in organizations and clubs^30^. Respondents were asked if they had more or less than monthly contact with family, relatives, and friends (in person or by telephone or email), and whether they belonged to any clubs, churches or other organizations. Individuals who had less than monthly contact with any of these categories or did not belong to any clubs, churches or organizations were classified as isolated. Number of close relationships was determined by self-report, and loneliness using the short form of the Revised UCLA loneliness scale^31^. Volunteering was assessed as a measure of prosocial behavior. Participants were asked whether they carried out any volunteer work, and those who volunteered at least once per month were classified as volunteers.

#### Health, disability and physical capability measures

Self-rated health was assessed on a 5-point rating from *excellent* to *poor*, and we analyzed the proportion of individuals giving fair/poor ratings^32^. Information about six doctor-diagnosed chronic diseases (coronary heart disease, stroke, cancer, diabetes, chronic lung disease and arthritis) was collected. Cross-sectional analyses were based on whether or not participants reported at least one chronic illness. In the longitudinal analyses, the average number of chronic illnesses (adjusted for baseline number) was analyzed. Participants were questioned about the presence of impairments in six activities of daily living (ADLs, e.g. difficulty in bathing or showering) that lasted at least 6 months. At baseline, the proportion with impaired ADLs was analyzed, while longitudinally the analysis was of incident ADL impairment among people with no baseline impairment. Gait speed was assessed with two 8-foot walking tests from a standing start by respondents aged ≥ 60 years; the test was not administered to younger participants. Participants were classified as obese if their body mass index was ≥ 30. Central obesity was measured as waist circumference, with the gender-specific cut-points recommended by the National Heart, Lung, and Blood Institute (102 cm for men, and 88 cm for women) being used to define central obesity.

### Statistical analysis

Associations between life skills and continuously distributed outcomes were analyzed using ordinary least squares (OLS) regression, while binary logistic regression was used to analyze the categorical outcomes, with the low skill group as the reference category. All models included age, sex, ethnicity, father’s education, own educational attainment and baseline cognition as covariates to ensure that associations between life skills and outcomes were not due to early socioeconomic endowments or current cognitive ability. Standardized β (*SE*) and odds ratios (OR, with 95% confidence intervals, CI) adjusted for covariates are presented, together with adjusted means or percentages in each category. Contrast analysis assessed linear gradients across the number of life skills.

#### Sensitivity analyses

We carried out four sets of sensitivity analysis. The first set tested the possibility that one of the five life skills contributing to the cumulative index (conscientiousness, for example) was primarily responsible for the findings. Analyses were therefore repeated after omitting each component in turn. Second, we explored the possibility that associations were driven by the effects of wealth or health. If a greater number of life skills is related to greater wealth or better health, it is possible that these factors are responsible for apparent associations between life skills and other outcomes. Baseline wealth quintile and self-rated health were therefore included as covariates. The third set of sensitivity tests repeated all analyses with a continuous life skill measure, in case the findings were an artefact of categorizing the skill components. We normalized scores across the complete distribution of each life skill, and averaged these scores for inclusion in the regression. Fourth, we tested whether the same pattern of results would emerge if analysis was restricted to the majority white ethnic group. There were insufficient respondents in the Black and Other groups to analyze them separately.

### Availability of data and ethical approval

The English Longitudinal Study of Ageing has been approved by the National Health Service Health Research Authority through the National Research Ethics Service. All methods were carried out in accordance with relevant guidelines and regulations, and participants provided informed consent. The data used in these analyses are available from the Gateway to Global Aging Data (https://g2aging.org/).

## Results

The sample comprised 3784 (42.8%) male and 5059 (57.2%) female participants in the HRS with an age range of 50–102 (*Mean* = 72.57, *SD* = 9.86) assessed in 2008/2010. The distribution of the five life skills is shown in Table 1: 2097 (23.7%) had low life skills with no attributes in the high skill category, 2605 (29.5%) had 1 skill, 2028 (22.9%) 2, 1226 (13.9%) 3, and 887 (10.0%) 4 or 5 life skills. People with more life skills were slightly younger on average (*p* < 0.001) with no differences between men and women. Higher scores on the life skills index were associated with higher levels of father’s education, own education, and greater cognitive ability (*P* < 0.001). Additionally, a greater proportion of black than white participants reported more life skills.

**Table 1:** Characteristics of life skill groups.

	Life skill groups					*p* *(linear contrast)*
	Low (n = 2097)	1 (n = 2605)	2 (n = 2028)	3 (n = 1226)	4/5 (n = 887)	
Age (years) mean ± SD	73.13 ± 10.34	72.97 ± 9.81	72.38 ± 9.70	71.51 ± 9.68	71.94 ± 9.28	<0.001
Gender, n (%)						
Men	907 (43.3)	1129 (43.3)	877 (43.2)	519 (42.3)	352 (39.7)	0.11
Women	1190 (56.7)	1476 (56.7)	1151 (56.8)	707 (57.7)	535 (60.3)	
Ethnicity, n (%)						
White	1811 (86.4)	2221 (85.3)	1677 (82.7)	997 (81.3)	718 (80.9)	<0.001
Black	200 (9.5)	283 (10.9)	275 (13.6)	178 (14.5)	131 (14.3)	
Other	86 (4.1)	101 (3.9)	76 (3.7)	51 (4.2)	38 (4.3)	
Father’s education n (%)						
Lower	632 (30.1)	732 (28.1)	567 (28.0)	279 (22.8)	216 (24.4)	<0.001
Intermediate	771 (36.8)	954 (36.6)	704 (34.7)	470 (38.3)	278 (31.3)	
Higher	694 (33.1)	919 (35.3)	757 (37.3)	477 (38.9)	393 (44.3)	
Educational qualifications, n (%)						
Less than high school	387 (18.5)	481 (18.5)	334 (16.5)	152 (12.4)	89 (10.0)	<0.001
High school	839 (40.0)	996 (38.2)	744 (36.7)	396 (32.3)	298 (33.6)	
Some college	468 (22.3)	596 (22.9)	480 (23.7)	308 (25.1)	223 (25.1)	
College and higher	403 (19.2)	532 (20.4)	470 (23.2)	370 (30.2)	277 (31.2)	
Baseline cognition (z scores)	22.49 ± 4.81	23.09 ± 4.61	23.51 ± 4.41	24.12 ± 4.39	24.77 ± 3.92	<0.001
mean ± SD						

There were positive associations between life skills, wealth and income (Table 2). The proportion of individuals in the low skill category with wealth and income in the highest quintile was 17.6% and 18.8% respectively, rising to 21.7% and 23.7% of those with 4/5 life skills. Compared with the low skill category (reference group), the odds of being in the high wealth quintile adjusted for age, sex, ethnicity, father’s education, own education and cognitive ability ranged from 1.20 (95%CI 1.01–1.41) for individuals with 1 life skill to 1.37 (95%CI 1.11–1.79) for those with 4/5 skills (full regression models are shown in supplementary Tables S1–S4). Similar results emerged when wealth and income were modeled as continuous variables. These findings confirm a graded relationship between number of life skills and economic success.

**Table 2:** Associations between life skills and economic prosperity, emotional and social outcomes.

	Life skill groups					*p (linear contrast)*
	Low	1	2	3	4/5	
Wealth (% in highest quintile) Adjusted OR (95% CI) N = 8416	17.6 (0.9) 1 (ref)	20.1 (0.8) 1.20 (1.01–1.41)	20.7 (0.9) 1.27 (1.07–1.51)	21.4 (1.1) 1.32 (1.09–1.60)	21.7 (1.3) 1.37 (1.11–1.68)	0.006
Income (% in highest quintile) Adjusted OR (95% CI) N = 8416	18.8 (0.8) 1 (ref)	18.7 (0.7) 1.01 (0.85–1.20)	20.7 (0.8) 1.18 (0.99–1.41)	20.8 (1.1) 1.17 (0.96–1.42)	23.9 (1.3) 1.45 (1.17–1.79)	<0.001
Depressive symptoms (% above threshold) Adjusted OR (95% CI) N = 8231	21.0 (0.7) 1 (ref)	12.2 (0.6) 0.51 (0.43–0.60)	8.1 (0.7) 0.32 (0.26–0.39)	4.6 (0.9) 0.15 (0.11–0.21)	0.42 (1.1) 0.12 (0.08–0.18)	<0.001
Anxiety (mean rating) Beta (SE) N = 8757	1.87 (0.01) Ref	1.62 (0.01) −0.197 (0.012)	1.44 (0.01) −0.311 (0.012)	1.32 (0.02) −0.330 (0.011)	1.22 (0.02) −0.340 (0.011)	<0.001
Financial strain (% above threshold) Adjusted OR (95% CI) N = 8677	35.7 (0.9) 1 (ref)	31.2 (0.8) 0.79 (0.69–0.90)	23.4 (0.9) 0.50 (0.43–0.58)	22.2 (1.2) 0.47 (0.39–0.55)	16.5 (1.4) 0.30 (0.24–0.37)	<0.001
Chronic stress (mean rating) Beta (SE) N = 4456	3.87 (0.09) Ref	3.22 (0.08) −0.096 (0.017)	2.61 (0.09) −0.172 (0.017)	2.23 (0.12) −0.182 (0.016)	1.74 (0.14) −0.206 (0.016)	<0.001
Social isolation (% isolated) Adjusted OR (95% CI) N = 6824	19.5 (0.9) 1 (ref)	17.9 (0.8) 0.91 (0.77–1.08)	14.4 (0.9) 0.71 (0.58–0.85)	15.0 (1.2) 0.74 (0.59–0.92)	13.1 (1.4) 0.61 (0.46–0.80)	<0.001
Loneliness (mean rating) Beta (SE) N = 8807	1.69 (0.01) 1 (ref)	1.52 (0.01) −0.148 (0.012)	1.35 (0.01) −0.270 (0.012)	1.26 (0.01) −0.283 (0.012)	1.19 (0.02) −0.284 (0.011)	<0.001
Close relationships (mean number) Beta (SE) N = 8794	8.38 (0.12) Ref	9.01 (0.10) 0.053 (0.013)	9.38 (0.12) 0.077 (0.013)	10.10 (0.15) 0.109 (0.012)	10.09 (0.18) 0.095 (0.012)	<0.001
Volunteering (% volunteering) Adjusted OR (95% CI) N = 8521	31.4 (1.0) 1 (ref)	33.4 (0.9) 1.11 (0.97–1.26)	38.3 (1.1) 1.37 (1.20–1.57)	40.9 (1.4) 1.53 (1.31–1.78)	42.3 (1.6) 1.61 (1.35–1.90)	<0.001

Note: Percentage or mean with standard error in parentheses. All values are adjusted for age, gender, ethnicity, father’s education, own education and baseline cognitive performance.

Emotional wellbeing was also positively associated with life skills; 21.0% of the low skill group had depressive symptoms above threshold, falling to 4.2% in the 4/5 skill group. The adjusted odds decreased from 0.51 (95%CI 0.43–0.60) for the 1 skill to 0.12 (95%CI 0.08–0.18) in the 4/5 skill group, indicating an 88% decrease in the odds of being depressed for people in the highest compared with low skill category. Anxiety scores declined progressively with greater numbers of life skills (*p* < 0.001); mean anxiety ratings were 75% lower in the 4/5 skill compared with the low skill group after adjustment for covariates. Two measures of stress (perceptions of financial strain and chronic stress ratings) were inversely associated with number of life skills. The proportion of participants reporting financial strain decreased progressively across life skill categories, with 50% fewer in the 4/5 skill than low skill groups. Mean ratings of chronic stress from family, housing and other sources fell from 3.87 in the low skill to 2.23 and 1.74 in the 3 and 4/5 skill groups, with significant gradients across the life skill categories in fully adjusted models (*P* < 0.001).

In the social domain, number of life skills was inversely associated with social isolation and loneliness (Table 2). The adjusted odds of social isolation were 26% lower in the 3 skill and 39% lower in the 4/5 skill compared with the reference category. Mean loneliness ratings were 1.69 in the low skill group, falling to 1.52 (β = −0.148) in the 1 skill, down to 1.19 (β = −0.284) in the 4/5 skill category. Conversely, life skills were positively related to having more close relationships and to greater prosocial behavior, after adjustment for covariates. Interestingly, 42.3% of the 4/5 skill participants had volunteered to work with youth or charities at least once in the last month, compared with 31.4% of the low skill group, representing a 61% greater adjusted odds in the highest skill category.

Results of analyses relating life skills with health, disability, physical capability and adiposity are summarized in Table 3 (full models in supplementary Tables S5–S7). Two aspects of health were included in these analyses. The proportion of participants reporting fair or poor self-rated health was strongly associated with number of life skills, decreasing across skill groups from 33.5% in the low to 13.9% in the 4/5 skill group. This was supported by the marked gradient in adjusted odds of being in fair or poor self-rated health, falling from 0.73 (95%CI 0.64–0.83) to 0.26 (95%CI 0.21–0.34) between the 1 and 4/5 skill categories. Overall, the proportion of respondents with one or more chronic illnesses was high (averaging 81.2%), and ranged from 84.8% in the low skill to 75.8% in the highest skill categories. The odds of having a chronic illness was significantly reduced in all skill groups compared with the low life skill group, with a progressive decline with accumulating life skills. Number of life skills was also inversely associated with the presence of one or more impaired ADLs. Nearly one quarter of the low skill group (24.3%) reported impaired ADLs, falling to 13.4% in the 3 and 10.4% in the 4/5 skill category. The adjusted odds were substantially lowered in all life skill groups compared with the low skill category, decreasing from 0.69 for 1 skill to 0.31 in the 4/5 skill category. These findings were corroborated with objectively measured walking or gait speed, which was significantly faster among respondents with greater numbers of life skills.

**Table 3:** Associations between life skills and health, disability, physical capability, and adiposity.

	Life skill groups					*p(linear contrast)*
	Low	1	2	3	4/5	
Self-rated health (% with fair/poor health) Adjusted OR (95% CI) N = 8413	33.5 (0.9) 1 (ref)	27.2 (0.8) 0.73 (0.64–0.83)	21.6 (0.9) 0.52 (0.45–0.60)	18.2 (1.2) 0.40 (0.33–0.49)	13.9 (1.4) 0.26 (0.21–0.34)	<0.001
Chronic illness (% with illness) Adjusted OR (95% CI) N = 8841	84.8 (0.8) 1 (ref)	82.4 (0.7) 0.80 (0.68–0.94)	81.1 (0.8) 0.72 (0.60–0.85)	79.7 (1.1) 0.65 (0.54–0.79)	75.8 (1.2) 0.51 (0.41–0.63)	<0.001
Activities of daily living (% impaired ADL) Adjusted OR (95% CI) N = 8843	24.3 (0.8) 1 (ref)	18.2 (0.7) 0.69 (0.60–0.80)	14.6 (0.8) 0.52 (0.44–0.61)	13.4 (1.0) 0.45 (0.37–0.55)	10.4 (1.2) 0.31 (0.24–0.41)	<0.001
Gait speed (mean in m/s) Beta (SE) N = 5899	0.737 (0.006)Ref	0.753 (0.005) 0.031 (0.014)	0.757 (0.006) 0.040 (0.014)	0.781 (0.008) 0.065 (0.013)	0.794 (0.009) 0.074 (0.013)	<0.001
Obesity (% obese) Adjusted OR (95% CI) N = 8274	31.5 (1.0) 1 (ref)	30.3 (0.9) 0.94 (0.82–1.07)	30.8 (1.0) 0.95 (0.82–1.09)	28.9 (1.3) 0.86 (0.73–1.01)	25.0 (1.6) 0.70 (0.58–0.85)	<0.001
Waist circumference (% above threshold) Adjusted OR (95% CI) N = 7655	68.4 (1.1) 1 (ref)	67.4 (1.0) 0.95 (0.83–1.09)	65.5 (1.1) 0.87 (0.75–0.99)	65.5 (1.4) 0.87 (0.74–1.02)	62.5 (1.7) 0.76 (0.63–0.91)	0.002

Note: Percentage or mean with standard error in parentheses. All values are adjusted for age, gender, ethnicity, father’s education, own education and baseline cognitive performance.

General obesity (body mass index ≥ 30) and central obesity (waist circumference ≥ sex-specific cut-points) were both inversely associated with number of life skills. The proportion of respondents who were obese was 31.5% in the low skill group, falling to 25.0% in the 4/5 skill group after adjustment of covariates, with a 30% reduction in adjusted odds in the highest compared with the low skill group. The number of respondents with waist circumferences indicative of central obesity was high, with the proportions being 62.5% in the 4/5 skill category, 65.5% in the 2 and 3 skill categories, rising to 68.4% among low skill participants (*P* = 0.002).

### Longitudinal analyses

These cross-sectional analyses cannot determine temporal relationships, so we assessed longitudinal associations between life skills measured in 2008/2010 and outcomes assessed in 2014. These results are summarized in Table 4, with full regression models detailed in supplementary Tables S8–S14. There was no significant relationship between life skills and wealth or income 4 years later, once baseline wealth or income had been taken into account. This indicates that the differences across life skill groups observed in the cross-sectional analyses were not augmented over time. However, longitudinal associations were observed between life skills and depressive symptoms in 2014 and anxiety in 2012. After adjustment for baseline depression and other covariates, the proportion of respondents with depressive symptoms above threshold declined progressively from 18.2% among people with low skills to 7.7% in the 4/5 skill group, reflecting a 75% decrease in adjusted odds. Anxiety ratings also decreased with more life skills after adjustment for covariates including baseline anxiety (*P* < 0.001). The two stress measures showed similar patterns. Even though differences were less marked than in cross-sectional analyses, the proportion of people experiencing financial strain was significantly higher in the low than the higher life skill groups. Mean ratings of chronic stress were also higher in the low skill group, indicating that the differences observed at baseline may be exacerbated over time.

**Table 4:** Associations between life skills in 2008/10 and social and health outcomes in 2014.

	Life skill groups					*p(linear contrast)*
	Low	1	2	3	4/5	
Depressive symptoms (% above threshold)^1^ Adjusted OR (95% CI) N = 6579	18.2 (0.8) 1 (ref)	13.6 (0.7) 0.69 (0.56–0.84)	10.1 (0.8) 0.45 (0.36–0.57)	8.4 (1.0) 0.32 (0.23–0.44)	7.7 (1.1) 0.25 (0.16–0.37)	<0.001
Anxiety (mean rating)^2^ Beta (SE) N = 2754	1.62 (0.02) Ref	1.61 (0.02) −0.006 (0.021)	1.49 (0.02) −0.096 (0.021)	1.47 (0.02) −0.092 (0.020)	1.43 (0.03) −0.105 (0.019)	<0.001
Financial strain (% above threshold)^3^ Adjusted OR (95% CI) N = 3518	28.0 (1.0) 1 (ref)	26.0 (1.1) 0.85 (0.67–1.09)	24.5 (1.2) 0.77 (0.59–0.99)	20.1 (1.6) 0.51 (0.37–0.71)	22.4 (1.9) 0.61 (0.41–0.92)	0.001
Chronic stress (mean rating)^4^ Beta (SE) N = 3076	3.49 (0.10) Ref	3.12 (0.09) −0.053 (0.019)	2.83 (0.10) −0.090 (0.019)	2.68 (0.13) −0.093 (0.018)	2.71 (0.15) −0.077 (0.018)	<0.001
Loneliness (mean rating)^5^ Beta (SE) N = 3651	1.53 (0.02) Ref	1.47 (0.01) −0.050 (0.018)	1.41 (0.02) −0.094 (0.018)	1.36 (0.02) −0.115 (0.017)	1.34 (0.02) −0.108 (0.016)	<0.001
Close relationships (mean number)^6^ Beta (SE) N = 3566	8.51 (0.15) Ref	8.83 (0.14) 0.027 (0.018)	9.22 (0.15) 0.057 (0.018)	9.26 (0.20) 0.050 (0.017)	9.26 (0.23) 0.044 (0.016)	0.001
Volunteering (% stopping volunteering)^7^ Adjusted OR (95% CI) N = 1338	41.4 (2.9) 1 (ref)	35.8 (2.5) 0.79 (0.56–1.10)	35.0 (2.6) 0.78 (0.55–1.10)	29.0 (3.2) 0.57 (0.38–0.84)	28.2 (3.6) 0.54 (0.35–0.84)	0.001
Self-rated health (% deterioration)^8^ Adjusted OR (95% CI) N = 5417	21.4 (1.1) 1 (ref)	17.1 (0.9) 0.76 (0.62–0.93)	14.5 (1.0) 0.63 (0.50–0.78)	11.8 (1.2) 0.47 (0.36–0.62)	11.0 (1.4) 0.43 (0.32–0.58)	<0.001
Chronic illnesses (mean number)^9^ Beta (SE) N = 7835	1.81 (0.013) Ref	1.80 (0.011) −0.007 (0.007)	1.77 (0.013) −0.015 (0.006)	1.79 (0.016) −0.006 (0.006)	1.73 (0.019) −0.021 (0.006)	0.001
Activities of daily living (incident impaired ADL %)^10^ Adjusted OR (95% CI) N = 7309	13.2 (0.8) 1 (ref)	11.4 (0.07) 0.84 (0.69–1.03)	10.9 (0.7) 0.80 (0.65–0.99)	9.4 (0.9) 0.67 (0.52–0.87)	7.5 (1.1) 0.51 (0.38–0.70)	<0.001
Gait speed (mean in m/s)^11^ Beta (SE) N = 2045	0.708 (0.009) Ref	0.723 (0.007) 0.032 (0.022)	0.739 (0.008) 0.060 (0.022)	0.746 (0.011) 0.060 (0.020)	0.741 (0.012) 0.046 (0.020)	0.006

Note: Percentage or mean with standard error in parentheses. All values are adjusted for age, gender, ethnicity, father’s education, own education and baseline cognitive performance.

^1^Additional adjustment for baseline depressive symptoms; ^2^Additional adjustment for baseline; anxiety measured in 2012; ^3^Additional adjustment for baseline financial strain; ^4^Additional adjustment for baseline chronic stress; ^5^Additional adjustment for baseline loneliness; ^6^Additional adjustment for baseline close relationships; ^7^Analysis restricted to participants who were volunteering at baseline; ^8^Proportion of participants with good to excellent health at baseline and fair or poor health on follow-up; ^9^Additional adjustment for baseline number of chronic illnesses; ^10^Analysis restricted to participants with no impaired ADLs at baseline; ^11^Additional adjustment for baseline gait speed.

Table 4 also shows that loneliness on follow-up was inversely associated with life skills at baseline independently of loneliness at baseline and other covariates. Conversely, the number of close relationships on follow-up was positively associated with life skills at baseline. In relation to prosocial behavior, we carried out separate analyses of the proportion of respondents who started volunteering between 2008/2010 and 2014, and the proportion of existing volunteers who had stopped volunteering on follow up. There was no association between life skills and taking up volunteering over this period. However, respondents with more life skills were less likely to stop volunteering; out of the 1,338 volunteers at baseline, 29% of those with 3 skills and 28.2% of those with 4/5 skills had stopped in 2014, compared with 41.4% of those with low and 35.8% with 1 life skill. So life skills were not associated with the likelihood of taking up volunteering over this 4 year period of middle and older age, but skills did predict whether existing volunteers had stopped volunteering on follow-up.

Changes in self-rated health were analyzed as the proportion of respondents in good to excellent self-rated health at baseline who had deteriorated to fair/poor health in 2014, and the mean number of chronic illnesses in 2014, adjusted for baseline levels. The results in Table 4 indicate that both measures were associated with life skills in a graded fashion. The proportion of participants whose health had deteriorated over this 4 year period was substantial, with 21.4% of the low skills group moving into the fair or poor health category, compared with just 11.0% in the 4/5 skill group. This represents a 57% reduction in adjusted odds in the highest skill group. The number of chronic illnesses also fell with increasing numbers of life skills, controlling for baseline chronic illness. We found that the incidence of impaired ADLs over the follow-up period among individuals with no impaired ADLs at baseline was inversely associated with life skills. Some 13.2% of the low skill group developed one or more impaired ADLs compared with only 7.8% of the 4/5 skill group, and the association was graded over the three intermediate categories. The faster gait speed in people with more life skills observed at baseline was sustained on follow-up even after adjusting for baseline gait speed (*P* = 0.006). However, there were no significant associations between life skills and the two measures of adiposity on follow-up after baseline measures had been taken into account.

### Sensitivity analyses

The first sensitivity analysis involved repeating the modeling after omitting each component of the life skills index in turn. The results are summarized in Table 5, where the adjusted ORs/betas for the 4/5 skill compared with the low skill group are presented along with *P* values for linear gradients across life skill categories. It is evident that associations were largely maintained when different components were omitted. The ORs/betas were reduced in some cases, but only two of the associations observed with the full index became non-significant. Since there were 135 analyses, these may be chance effects.

**Table 5:** Sensitivity analyses: life skill index excluding each component in turn.

	Full index		Excluding conscientiousness		Excluding emotional stability		Excluding persistence		Excluding optimism		Excluding control	
	*OR, β* ^1^	*p* ^2^	*OR, β*	*p*	*OR, β*	*p*	*OR, β*	*p*	*OR, β*	*p*	*OR, β*	*p*
Wealth	1.37	0.006	1.32	0.010	1.31	0.009	1.35	0.001	1.30	0.003	1.24	0.036
Income	1.45	<0.001	1.38	0.001	1.43	<0.001	1.27	0.002	1.35	<0.001	1.35	0.001
Depressive symptoms	0.12	<0.001	0.12	<0.001	0.17	<0.001	0.13	<0.001	0.14	<0.001	0.18	<0.001
Anxiety	−0.340	<0.001	−0.381	<0.001	−0.303	<0.001	−0.343	<0.001	−0.390	<0.001	−0.306	<0.001
Financial strain	0.30	<0.001	0.36	<0.001	0.37	<0.001	0.35	<0.001	0.35	<0.001	0.43	<0.001
Chronic stress	−0.206	<0.001	−0.229	<0.001	−0.193	<0.001	−0.245	<0.001	−0.230	<0.001	−0.176	<0.001
Social isolation	0.61	<0.001	0.622	<0.001	0.64	<0.001	0.71	<0.001	0.67	<0.001	0.69	0.001
Loneliness	0.12	<0.001	0.14	<0.001	0.17	<0.001	0.15	<0.001	0.16	<0.001	0.20	<0.001
Close relationships	0.095	<0.001	0.120	<0.001	0.109	<0.001	0.110	<0.001	0.109	<0.001	0.081	<0.001
Volunteering	1.61	<0.001	1.57	<0.001	1.52	<0.001	1.56	<0.001	1.53	<0.001	1.56	<0.001
Self−rated health	0.26	<0.001	0.32	<0.001	0.35	<0.001	0.29	<0.001	0.33	<0.001	0.37	<0.001
Chronic disease	0.51	<0.001	0.61	<0.001	0.58	<0.001	0.54	<0.001	0.59	<0.001	0.60	<0.001
Impaired ADLs	0.31	<0.001	0.40	<0.001	0.40	<0.001	0.33	<0.001	0.37	<0.001	0.43	<0.001
Gait speed	0.074	<0.001	0.079	<0.001	0.079	<0.001	0.083	<0.001	0.084	<0.001	0.061	<0.001
Waist circumference	0.76	<0.001	0.81	0.013	0.75	<0.001	0.82	0.008	0.82	0.006	0.81	0.004
Obesity	0.70	<0.001	0.82	0.079	0.75	<0.001	0.76	0.001	0.79	0.003	0.72	<0.001
**Longitudinal results**												
Depressive symptoms	0.25	<0.001	0.25	<0.001	0.40	<0.001	0.23	<0.001	0.28	<0.001	0.29	<0.001
Anxiety	−0.105	<0.001	−0.136	<0.001	−0.100	<0.001	−0.132	<0.001	−0.116	<0.001	−0.080	<0.001
Financial strain	0.61	0.001	0.53	<0.001	0.60	0.001	0.55	<0.001	0.58	<0.001	0.68	0.012
Chronic stress	−0.077	<0.001	−0.086	<0.001	−0.088	<0.001	−0.106	<0.001	−0.096	<0.001	−0.057	<0.001
Loneliness	−0.108	<0.001	−0.118	<0.001	−0.107	<0.001	−0.144	<0.001	−0.125	<0.001	−0.095	<0.001
Close relationships	0.044	0.001	0.049	<0.001	0.048	0.001	0.055	0.001	0.046	0.001	0.046	0.003
Volunteering	0.54	0.001	0.58	0.001	0.59	0.001	0.51	0.001	0.60	0.003	0.59	<0.001
Self−rated health	0.43	<0.001	0.48	<0.001	0.49	<0.001	0.44	<0.001	0.46	<0.001	0.48	<0.001
Chronic disease	−0.021	0.001	−0.019	0.003	−0.014	0.011	−0.019	0.001	−0.019	0.002	−0.018	0.006
Impaired ADLs	0.51	<0.001	0.55	<0.001	0.67	<0.001	0.59	<0.001	0.55	<0.001	0.59	<0.001
Gait speed	0.046	0.006	0.058	0.001	0.047	0.014	0.057	0.003	0.067	0.001	0.038	0.066

^1^Adjusted odds ratio (OR) for the highest life skill category, or standardized regression coefficient *β* for continuously distributed outcomes. Results for continuously distributed variables are shown with 3 decimal points, and OR with 2 points. All analyses are adjusted for age, sex, ethnicity, father’s education, own education, and cognitive function.

^2^*P* is for linear gradients across life skill categories.

The second set of sensitivity analyses tested the possibility that associations were driven by wealth or health. The results in Table 6 indicate that both the cross-sectional and longitudinal associations between life skills and economic, psychological, social, health and disability outcomes were maintained after wealth and health had been taken into account. Third, we repeated all analyses with a continuous instead of a categorical life skill measure. The results were unchanged except for the loss of 3 associations: the longitudinal analyses of volunteering, number of chronic diseases, and gait speed (Table 6). The final sensitivity tests repeated analysis on the majority white ethnic group only. The pattern of results was unchanged (Table 6), even though the sample size was reduced.

**Table 6:** Sensitivity analyses.

	Full index		Wealth and self−rated health covariates		Continuous life skills index		White participants	
	*OR, β* ^1^	*p* ^2^	*OR, β*	*p*	*OR, β*	*p*	*OR, β*	*p*
Wealth	1.37	0.006			1.25	<0.001	1.40	0.002
Income	1.45	<0.001	1.23	0.027	1.37	<0.001	1.37	0.002
Depressive symptoms	0.12	<0.001	0.16	<0.001	0.44	<0.001	0.01	<0.001
Anxiety	−0.340	<0.001	−0.309	<0.001	−0.184	<0.001	−0.346	<0.001
Financial strain	0.30	<0.001	0.33	<0.001	0.64	<0.001	0.27	<0.001
Chronic stress	−0.206	<0.001	−0.183	<0.001	−0.114	<0.001	−0.218	<0.001
Social isolation	0.61	<0.001	0.65	<0.001	0.64	<0.001	0.62	<0.001
Loneliness	0.12	<0.001	0.13	<0.001	0.43	<0.001	0.10	<0.001
Close relationships	0.095	<0.001	0.088	<0.001	0.136	<0.001	0.093	<0.001
Volunteering	1.61	<0.001	1.50	<0.001	1.52	<0.001	1.60	<0.001
Self−rated health	0.26	<0.001	—		0.52	<0.001	0.23	<0.001
Chronic disease	0.51	<0.001	0.63	<0.001	0.79	<0.001	0.55	<0.001
Impaired ADLs	0.31	<0.001	0.43	<0.001	0.58	<0.001	0.30	<0.001
Gait speed	0.074	<0.001	0.059	<0.001	0.101	<0.001	0.074	<0.001
Waist circumference	0.76	<0.001	0.81	0.035	0.80	<0.001	0.74	0.002
Obesity	0.70	<0.001	0.79	0.028	0.82	<0.001	0.67	<0.001
**Longitudinal results**								
Depressive symptoms	0.25	<0.001	0.29	<0.001	0.60	<0.001	0.18	<0.001
Anxiety	−0.105	<0.001	−0.098	<0.001	−0.050	0.002	−0.104	<0.001
Financial strain	0.61	0.001	0.64	0.002	0.72	<0.001	0.56	<0.001
Chronic stress	−0.077	<0.001	−0.071	<0.001	−0.070	<0.001	−0.086	<0.001
Loneliness	−0.108	<0.001	−0.101	<0.001	−0.068	<0.001	−0.110	<0.001
Close relationships	0.044	0.001	0.038	0.009	0.059	<0.001	0.041	0.013
Volunteering	0.54	0.001	0.59	0.005	0.81	0.12	0.48	0.001
Self−rated health	0.43	<0.001	—		0.62	<0.001	0.62	0.007
Chronic disease	−0.021	0.001	−0.019	0.006	−0.009	0.093	−0.027	<0.001
Impaired ADLs	0.51	<0.001	0.55	<0.001	0.79	0.003	0.52	<0.001
Gait speed	0.046	0.006	0.034	0.038	0.023	0.19	0.058	0.001

^1^Adjusted odds ratio (OR) for the highest life skill category, or standardized regression coefficient *β* for continuously distributed outcomes. Results for continuously distributed variables are shown with 3 decimal points, and OR with 2 points. All analyses are adjusted for age, sex, ethnicity (except for analyses of White participants), father’s education, own education, and cognitive function.

^2^*P* is for linear gradients across life skill categories.

## Discussion

These analyses of the HRS showed that among older Americans, greater numbers of life skills were associated with greater wealth and income, fewer depressive symptoms, less anxiety and stress, less social isolation and loneliness, more close relationships and volunteering, better self-rated health, less chronic illness and impaired ADLs, faster walking speed, and less obesity and central adiposity. Longitudinally over a 4 year period, more life skills were related to less depression, anxiety, stress and loneliness, more close relationships and volunteering, better health, less incident impaired ADLs, and faster walking speed, controlling for baseline levels.

The results confirm and extend findings from older people in England analyzed from ELSA^21^. The same 5 life skills were assessed, although the HRS had a more robust measure of optimism compared with ELSA. Additional outcome measures in HRS included anxiety, financial strain and chronic stress that were not available in ELSA. Conversely, ELSA included biomarkers such as C-reactive protein and vitamin D that were not measured in HRS. The cross-sectional results in the two analyses were identical. In neither case were there longitudinal associations between life skills and economic variables or adiposity, though both showed relationships with emotional, social and health outcomes.

The sensitivity analyses addressed a number of possible explanations of the findings. A plausible explanation is that one of the life skills such as conscientiousness was particularly important, and was responsible for the association between the cumulative index and outcomes. Yet when each attribute was removed in turn from the index, the findings did not change substantially (Table 5). The 5 skills included in these analyses may not be equally influential, and it is possible that smaller combinations of these attributes are important^33,34^. But we decided against repeating the analyses removing two or three skills at a time because of the multiple combinations that would need to be evaluated. We also considered whether the findings could be attributed to one of the outcome variables that was in turn associated with the other outcomes. In the light of well-established socioeconomic influences on health and wellbeing, we tested the impact of wealth as an index of socioeconomic resources. Ill-health may also have profound effects on social function, psychological wellbeing and participation in the labor market, so we evaluated the impact of self-rated health. However, the results do not indicate that either wealth or health was responsible for the findings.

The HRS has a diverse ethnic composition. Although we adjusted statistically for ethnicity in all analyses, the pattern of associations between life skills and outcomes might vary across ethnic groups. Unfortunately, we could not conduct the analyses fully stratifying for ethnicity, since the white ethnic group was the only one with a sufficient sample size.

These associations were independent of sociodemographic factors, childhood socioeconomic status and baseline cognitive ability, endorsing the importance of character strengths and capabilities in older adults. The life skills were measured when participants were already at advanced ages, so it is not known whether they reflect skills that evolved in childhood and adolescence, or capabilities developed over adult life. Many of these characteristics show moderate between-person stability, even though mean levels may change over time through the influence of social roles, aging and other factors^35^. It would be interesting to track these factors in mid-life as well as childhood and adolescence.

The non-cognitive life skills literature highlights additional factors that were not measured here, including social skills, leadership and self-esteem^6,36^. The measure of sense of control in this study is related to perceived mastery and self-efficacy, and it would have been desirable to include an assessment of self-control as well. It is important to emphasize that the associations identified here are causal. Although we took account of current sociodemographics, cognitive and childhood socioeconomic status, and showed in sensitivity analyses that neither economic nor health differences underlie the findings, other unmeasured factors might be responsible. Additionally, the majority of outcomes were based on self-report so may be vulnerable to reporting bias.

There are, of course, many other factors that contribute to psychosocial, economic and health status at older ages including adult life occupation and income, negative life events, genetic and environmental determinants of health, marital status, and health behaviors. Neurocognitive function and capability are also crucial for the maintenance of health and wellbeing. Nonetheless, the results provide impetus for understanding how life skills can be fostered and maintained in later years for the benefit of society and older people themselves. The fact that similar relationships between life skills and outcomes at older ages have been observed both in the USA and UK suggests that the associations are quite robust. Our observation of a cumulate impact of life skills indicates that focus on a single attribute may not be appropriate, and that a broader approach to psychological and social capabilities is desirable. Whether programs can be developed that enhance these capabilities in middle and older age in a sustained fashion has yet to be established.

## Electronic supplementary material

Supplementary tables
