# Supplementary material for: The Life Skills of Older Americans: Association with Economic, Psychological, Social, and Health Outcomes
Source: Sci Rep. 2018 Jul 5;8:9669. doi: 10.1038/s41598-018-27909-w (PMC6033934; doi:10.1038/s41598-018-27909-w)
Supplement: Supplementary file 1 — Supplementary tables [file 41598_2018_27909_MOESM1_ESM.docx]

**The Life Skills of Older Americans:**

**Association with Economic, Psychological, Social, and Health Outcomes**

**Andrew Steptoe and Sarah E. Jackson**

**Supplementary Tables**

**Table S1 Associations of life skills with economic factors**

| **Wealth (% in top quintile)**  **(n = 8,416)** | | |  | **Income (% in top quintile)**  **(n = 8,416)** | | |
| --- | --- | --- | --- | --- | --- | --- |
| **Factor** | **Adjusted OR**  **(95% CI)** | **P** |  | **Factor** | **Adjusted OR**  **(95% CI)** | **P** |
| Age | 1.03 (1.02-1.04) | <0.001 |  | Age | 0.95 (0.94-0.96) | <0.001 |
| Sex | 0.81 (0.72-0.92) | <0.001 |  | Sex | 0.64 (0.56-0.72) | <0.001 |
| Ethnicity |  |  |  | Ethnicity |  |  |
| White (ref) | 1 |  |  | White (ref) | 1 |  |
| Black | 0.22 (0.16-0.30) | <0.001 |  | Black | 0.57 (0.46-0.71) | <0.001 |
| Other | 0.60 (0.42-0.87) | 0.007 |  | Other | 0.90 (0.66-1.22) | 0.49 |
| Parental education |  |  |  | Parental education |  |  |
| Lower (ref) | 1 |  |  | Lower (ref) | 1 |  |
| Intermediate | 1.29 (1.01-1.53) | 0.002 |  | Intermediate | 1.41 (1.18-1.69) | <0.001 |
| Higher | 1.78 (1.51-2.11) | <0.001 |  | Higher | 1.67 (1.40-1.99) | <0.001 |
| Education |  |  |  | Education |  |  |
| Less than high school (ref) | 1 |  |  | Less than high school (ref) | 1 |  |
| High school | 1.81 (1.41-2.33) | <0.001 |  | High school | 1.20 (1.48-2.67) | <0.001 |
| Some college | 2.45 (1.89-3.18) | <0.001 |  | Some college | 3.16 (2.34-4.26) | <0.001 |
| College and higher | 5.07 (3.92-6.57) | <0.001 |  | College and higher | 7.74 (5.74-10.42) | <0.001 |
| Cognition | 1.06 (1.04-1.08) | <0.001 |  | Cognition | 1.07 (1.05-1.09) | <0.001 |
| Life skills |  |  |  | Life skills |  |  |
| 0 (ref) | 1 |  |  | 0 (ref) | 1 |  |
| 1 | 1.20 (1.01-1.41) | 0.033 |  | 1 | 1.01 (0.85-1.20) | 0.94 |
| 2 | 1.27 (1.07-1.51) | 0.004 |  | 2 | 1.18 (0.99-1.41) | 0.063 |
| 3 | 1.32 (1.09-1.60) | 0.005 |  | 3 | 1.17 (0.96-1.42) | 0.13 |
| 4,5 | 1.37 (1.11-1.68) | 0.004 |  | 4,5 | 1.45 (1.17-1.79) | 0.001 |
| Linear trend across categories |  | 0.006 |  | Linear trend across categories |  | <0.001 |

**Table S2 Associations of life skills with emotional wellbeing**

| **Depressive symptoms (% above threshold)**  **(n = 8,234)** | | |  | **Anxiety (mean rating)**  **(n = 8,757)** | | |
| --- | --- | --- | --- | --- | --- | --- |
| **Factor** | **Adjusted OR**  **(95% CI)** | **P** |  | **Factor** | **Beta**  **(SE)** | **P** |
| Age | 0.98 (0.97-0.99) | <0.001 |  | Age | -0.043 (0.010) | <0.001 |
| Sex | 1.86 (1.59-2.16) | <0.001 |  | Sex | 0.076 (0.010) | <0.001 |
| Ethnicity |  |  |  | Ethnicity |  |  |
| White (ref) | 1 |  |  | black vs white | 0.056 (0.010) | <0.001 |
| Black | 1.12 (0.91-1.39) | 0.73 |  | other vs white | 0.028 (0.01) | 0.005 |
| Other | 1.31 (0.95-1.80) | 0.098 |  | Parental education |  |  |
| Parental education |  |  |  | intermediate v lower | -0.022 (0.012) | 0.072 |
| Lower (ref) | 1 |  |  | higher v lower | -0.042 (0.013) | 0.001 |
| Intermediate | 0.83 (0.69-0.98) | 0.032 |  | Education |  |  |
| Higher | 0.73 (0.60-0.89) | 0.002 |  | High school v less than HS | -0.060 (0.015) | <0.001 |
| Education |  |  |  | Some college v less than HS | -0.069 (0.014) | <0.001 |
| Less than high school (ref) | 1 |  |  | College and higher v less than HS | -0.080 (0.015) | <0.001 |
| High school | 0.63 (0.52-0.77) | <0.001 |  | Cognition | -0.123 (0.011) | <0.001 |
| Some college | 0.67 (0.53-0.84) | <0.001 |  | Life skills |  |  |
| College and higher | 0.56 (0.43-0.73) | <0.001 |  | 1 vs 0 | -0.197 (0.012) | <0.001 |
| Cognition | 0.92 (0.90-0.94) | <0.001 |  | 2 vs 0 | -0.311 (0.012) | <0.001 |
| Life skills |  |  |  | 3 vs 0 | -0.033 (0.011) | <0.001 |
| 0 (ref) | 1 |  |  | 4,5 vs 0 | -0.034 (0.011) | <0.001 |
| 1 | 0.51 (0.43-0.60) | <0.001 |  | Linear trend across categories |  | <0.001 |
| 2 | 0.32 (0.26-0.39) | <0.001 |  |  |  |  |
| 3 | 0.15 (0.11-0.21) | <0.001 |  |  |  |  |
| 4,5 | 0.12 (0.08-0.18) | <0.001 |  |  |  |  |
| Linear trend across categories |  | <0.001 |  |  |  |  |

**Table S3 Associations of life skills with stress ratings**

| **Financial strain (% above threshold)**  **(n = 8,677)** | | |  | **Chronic stress (mean rating)**  **(n = 4,456)** | | |
| --- | --- | --- | --- | --- | --- | --- |
| **Factor** | **Adjusted OR**  **(95% CI)** | **P** |  | **Factor** | **Beta**  **(SE)** | **P** |
| Age | 0.95 (0.94-0.95) | <0.001 |  | Age | -0.291 (0.015) | <0.001 |
| Sex | 1.29 (1.16-1.43) | <0.001 |  | Sex | -0.059 (0.014) | <0.001 |
| Ethnicity |  |  |  | Ethnicity |  |  |
| White (ref) | 1 |  |  | black vs white | 0.071 (0.015) | <0.001 |
| Black | 2.00 (1.73-2.32) | <0.001 |  | other vs white | 0.007 (0.015) | 0.61 |
| Other | 1.11 (0.87-1.43) | 0.39 |  | Parental education |  |  |
| Parental education |  |  |  | intermediate v lower | 0.006 (0.018) | 0.72 |
| Lower (ref) | 1 |  |  | higher v lower | -0.004 (0.019) | 0.82 |
| Intermediate | 0.81 (0.71-0.92) | 0.001 |  | Education |  |  |
| Higher | 0.75 (0.65-0.86) | <0.001 |  | High school v less than HS | -0.006 (0.022) | 0.79 |
| Education |  |  |  | Some college v less than HS | 0.039 (0.022) | 0.074 |
| Less than high school (ref) | 1 |  |  | College and higher v less than HS | -0.013 (0.023) | 0.58 |
| High school | 0.73 (0.63-0.84) | <0.001 |  | Cognition | -0.045 (0.016) | 0.005 |
| Some college | 0.68 (0.57-0.80) | <0.001 |  | Life skills |  |  |
| College and higher | 0.41 (0.34-0.49) | <0.001 |  | 1 vs 0 | -0.096 (0.017) | <0.001 |
| Cognition | 0.96 (0.95-0.97) | <0.001 |  | 2 vs 0 | -0.172 (0.017) | <0.001 |
| Life skills |  |  |  | 3 vs 0 | -0.182 (0.016) | <0.001 |
| 0 (ref) | 1 |  |  | 4,5 vs 0 | -0.206 (0.016) | <0.001 |
| 1 | 0.79 (0.69-0.90) | <0.001 |  | Linear trend across categories |  | <0.001 |
| 2 | 0.50 (0.43-0.58) | <0.001 |  |  |  |  |
| 3 | 0.47 (0.39-0.55) | <0.001 |  |  |  |  |
| 4,5 | 0.30 (0.24-0.37) | <0.001 |  |  |  |  |
| Linear trend across categories |  | <0.001 |  |  |  |  |

**Table S4a Associations of life skills with social factors**

| **Social isolation (% isolated)**  **(n = 6,824)** | | |  | **Loneliness (mean rating)**  **(n = 8,807)** | | |
| --- | --- | --- | --- | --- | --- | --- |
| **Factor** | **Adjusted OR**  **(95% CI)** | **P** |  | **Factor** | **Beta**  **(SE)** | **P** |
| Age | 1.00 (0.99-1.00) | 0.037 |  | Age | -0.066 (0.011) | <0.001 |
| Sex | 0.60 (0.53-0.67) | <0.001 |  | Sex | 0.064 (0.010) | <0.001 |
| Ethnicity |  |  |  | Ethnicity |  |  |
| White (ref) | 1 |  |  | black vs white | 0.043 (0.010) | <0.001 |
| Black | 0.58 (0.46-0.74) | <0.001 |  | other vs white | 0.001 (0.010) | 0.99 |
| Other | 0.90 (0.65-1.26) | 0.54 |  | Parental education |  |  |
| Parental education |  |  |  | intermediate v lower | -0.010 (0.012) | 0.43 |
| Lower (ref) | 1 |  |  | higher v lower | -0.031 (0.013) | 0.022 |
| Intermediate | 1.05 (0.89-1.24) | 0.56 |  | Education |  |  |
| Higher | 0.83 (0.69-1.00) | 0.048 |  | High school v less than HS | 0.001 (0.015) | 0.98 |
| Education |  |  |  | Some college v less than HS | -0.013 (0.015) | 0.37 |
| Less than high school (ref) | 1 |  |  | College and higher v less than HS | -0.021 (0.015) | 0.17 |
| High school | 1.05 (0.86-1.27) | 0.64 |  | Cognition | -0.108 (0.012) | <0.001 |
| Some college | 0.95 (0.76-1.18) | 0.64 |  | Life skills |  |  |
| College and higher | 0.87 (0.68-1.10) | 0.25 |  | 1 vs 0 | -0.148 (0.012) | <0.001 |
| Cognition | 0.93 (0.92-0.95) | <0.001 |  | 2 vs 0 | -0.270 (0.012) | <0.001 |
| Life skills |  |  |  | 3 vs 0 | -0.283 (0.012) | <0.001 |
| 0 (ref) | 1 |  |  | 4,5 vs 0 | -0.284 (0.011) | <0.001 |
| 1 | 0.91 (0.77-1.08) | 0.27 |  | Linear trend across categories |  | <0.001 |
| 2 | 0.71 (0.58-0.85) | <0.001 |  |  |  |  |
| 3 | 0.74 (0.59-0.92) | 0.007 |  |  |  |  |
| 4,5 | 0.61 (0.46-0.80) | <0.001 |  |  |  |  |
| Linear trend across categories |  | <0.001 |  |  |  |  |

**Table S4b Associations of life skills with social factors**

| **Volunteering (% volunteering)**  **(n = 8,521)** | | |  | **Close relationships (mean number)**  **(n = 8,794)** | | |
| --- | --- | --- | --- | --- | --- | --- |
| **Factor** | **Adjusted OR**  **(95% CI)** | **P** |  | **Factor** | **Beta**  **(SE)** | **P** |
| Age | 1.00 (1.00-1.00) | 0.73 |  | Age | 0.072 (0.011) | <0.001 |
| Sex | 1.20 (1.09-1.32) | <0.001 |  | Sex | 0.053 (0.011) | <0.001 |
| Ethnicity |  |  |  | Ethnicity |  |  |
| White (ref) | 1 |  |  | black vs white | 0.080 (0.011) | <0.001 |
| Black | 1.51 (1.30-1.75) | <0.001 |  | other vs white | 0.026 (0.011) | 0.015 |
| Other | 1.10 (0.85-1.41) | 0.48 |  | Parental education |  |  |
| Parental education |  |  |  | intermediate v lower | -0.003 (0.013) | 0.83 |
| Lower (ref) | 1 |  |  | higher v lower | -0.021 (0.014) | 0.13 |
| Intermediate | 1.04 (0.92-1.18) | 0.52 |  | Education |  |  |
| Higher | 1.16 (1.02-1.32) | 0.022 |  | High school v less than HS | -0.060 (0.016) | <0.001 |
| Education |  |  |  | Some college v less than HS | -0.088 (0.016) | <0.001 |
| Less than high school (ref) | 1 |  |  | College and higher v less than HS | -0.119 (0.016) | <0.001 |
| High school | 1.32 (1.13-1.55) | 0.001 |  | Cognition | 0.057 (0.012) | <0.001 |
| Some college | 1.59 (1.34-1.89) | <0.001 |  | Life skills |  |  |
| College and higher | 2.22 (1.86-2.65) | <0.001 |  | 1 vs 0 | 0.053 (0.013) | <0.001 |
| Cognition | 1.07 (0.97-1.26) | <0.001 |  | 2 vs 0 | 0.077 (0.013) | <0.001 |
| Life skills |  |  |  | 3 vs 0 | 0.109 (0.012) | <0.001 |
| 0 (ref) | 1 |  |  | 4,5 vs 0 | 0.095 (0.012) | <0.001 |
| 1 | 1.11 (0.97-1.26) | 0.13 |  | Linear trend across categories |  | <0.001 |
| 2 | 1.37 (1.20-1.57) | <0.001 |  |  |  |  |
| 3 | 1.53 (1.31-1.78) | <0.001 |  |  |  |  |
| 4,5 | 1.61 (1.35-1.90) | <0.001 |  |  |  |  |
| Linear trend across categories |  | <0.001 |  |  |  |  |

**Table S5 Associations of life skills with health outcomes**

| **Self-rated health (% fair/poor health)**  **(n = 8,413)** | | |  | **Chronic illness (%)**  **(n = 8,841)** | | |
| --- | --- | --- | --- | --- | --- | --- |
| **Factor** | **Adjusted OR**  **(95% CI)** | **P** |  | **Factor** | **Adjusted OR**  **(95% CI)** | **P** |
| Age | 1.00 (1.00-1.01) | 0.37 |  | Age | 1.08 (1.07-1.09) | <0.001 |
| Sex | 1.11 (1.00-1.24) | 0.054 |  | Sex | 1.20 (1.07-1-.35) | 0.002 |
| Ethnicity |  |  |  | Ethnicity |  |  |
| White (ref) | 1 |  |  | White (ref) | 1 |  |
| Black | 1.35 (1.15-1.58) | <0.001 |  | Black | 1.08 (0.91-1.29) | 1.08 |
| Other | 1.68 (1.31-2.17) | <0.001 |  | Other | 0.70 (0.54-0.90) | 0.7 |
| Parental education |  |  |  | Parental education |  |  |
| Lower (ref) | 1 |  |  | Lower (ref) | 1 |  |
| Intermediate | 0.75 (0.66-0.85) | <0.001 |  | Intermediate | 1.03 (0.88-1.20) | 0.75 |
| Higher | 0.70 (0.61-0.81) | <0.001 |  | Higher | 0.92 (0.78-1.08) | 0.29 |
| Education |  |  |  | Education |  |  |
| Less than high school (ref) | 1 |  |  | Less than high school (ref) | 1 |  |
| High school | 0.62 (0.53-0.71) | <0.001 |  | High school | 0.99 (0.81-1.20) | 0.9 |
| Some college | 0.59 (0.50-0.70) | <0.001 |  | Some college | 0.97 (0.79-1.20) | 0.78 |
| College and higher | 0.43 (0.36-0.53) | <0.001 |  | College and higher | 0.71 (0.57-0.88) | 0.002 |
| Cognition | 0.92 (0.90-0.93) | <0.001 |  | Cognition | 1.00 (0.99-1.02) | 0.98 |
| Life skills |  |  |  | Life skills |  |  |
| 0 (ref) | 1 |  |  | 0 (ref) | 1 |  |
| 1 | 0.73 (0.64-0.83) | <0.001 |  | 1 | 0.80 (0.68-0.94) | 0.009 |
| 2 | 0.52 (0.45-0.60) | <0.001 |  | 2 | 0.72 (0.60-0.85) | <0.001 |
| 3 | 0.40 (0.33-0.49) | <0.001 |  | 3 | 0.65 (0.54-0.79) | <0.001 |
| 4,5 | 0.26 (0.21-0.34) | <0.001 |  | 4,5 | 0.51 (0.41-0.63) | <0.001 |
| Linear trend across categories |  | <0.001 |  | Linear trend across categories |  | <0.001 |

**Table S6 Associations of life skills with physical outcomes**

| **Activities of Daily Living (% impairment)**  **(n = 8,843)** | | |  | **Gait speed (m/s)**  **(n = 5,899)** | | |
| --- | --- | --- | --- | --- | --- | --- |
| **Factor** | **Adjusted OR**  **(95% CI)** | **P** |  | **Factor** | **Beta**  **(SE)** | **P** |
| Age | 1.04 (1.03-1.05) | <0.001 |  | Age | -0.256 (0.012) | <0.001 |
| Sex | 1.16 (1.03-1.31) | 0.015 |  | Sex | -0.139 (0.012) | <0.001 |
| Ethnicity |  |  |  | Ethnicity |  |  |
| White (ref) | 1 |  |  | black vs white | -0.155 (0.012) | <0.001 |
| Black | 1.37 (1.15-1.64) | <0.001 |  | other vs white | -0.022 (0.012) | 0.055 |
| Other | 1.47 (1.10-1.95) | 0.009 |  | Parental education |  |  |
| Parental education |  |  |  | intermediate v lower | 0.049 (0.014) | 0.001 |
| Lower (ref) | 1 |  |  | higher v lower | 0.071 (0.014) | <0.001 |
| Intermediate | 0.86 (0.75-0.99) | 0.037 |  | Education |  |  |
| Higher | 0.85 (0.72-1.00) | 0.046 |  | High school v less than HS | 0.056 (0.017) | 0.001 |
| Education |  |  |  | Some college v less than HS | 0.108 (0.017) | <0.001 |
| Less than high school (ref) | 1 |  |  | College and higher v less than HS | 0.133 (0.018) | <0.001 |
| High school | 0.78 (0.66-0.91) | 0.002 |  | Cognition | 0.180 (0.013) | <0.001 |
| Some college | 0.74 (0.61-0.89) | 0.001 |  | Life skills |  |  |
| College and higher | 0.64 (0.52-0.79) | <0.001 |  | 1 vs 0 | 0.031 (0.014) | 0.031 |
| Cognition | 0.93 (0.92-0.95) | <0.001 |  | 2 vs 0 | 0.040 (0.014) | 0.005 |
| Life skills |  |  |  | 3 vs 0 | 0.065 (0.013) | <0.001 |
| 0 (ref) | 1 |  |  | 4,5 vs 0 | 0.074 (0.013) | <0.001 |
| 1 | 0.69 (0.60-0.80) | <0.001 |  | Linear trend across categories |  | <0.001 |
| 2 | 0.52 (0.44-0.61) | <0.001 |  |  |  |  |
| 3 | 0.45 (0.37-0.55) | <0.001 |  |  |  |  |
| 4,5 | 0.31 (0.24-0.41) | <0.001 |  |  |  |  |
| Linear trend across categories |  | <0.001 |  |  |  |  |

**Table S7 Associations of life skills with adiposity**

| **Obesity (% BMI ≥30)**  **(n = 8,274)** | | |  | **Waist circumference (% above threshold)**  **(n = 7,655)** | | |
| --- | --- | --- | --- | --- | --- | --- |
| **Factor** | **Adjusted OR**  **(95% CI)** | **P** |  | **Factor** | **Adjusted OR**  **(95% CI)** | **P** |
| Age | 0.96 (0.95-0.96) | <0.001 |  | Age | 1.00 (0.99-1.00) | 0.23 |
| Sex | 1.05 (0.05-1.16) | 0.38 |  | Sex | 1.96 (1.78-2.17) | <0.001 |
| Ethnicity |  |  |  | Ethnicity |  |  |
| White (ref) | 1 |  |  | White (ref) | 1 |  |
| Black | 1.54 (1.33-1.79) | <0.001 |  | Black | 1.1.5 (0.98-1.35) | 0.098 |
| Other | 0.77 (0.60-1.00) | 0.05 |  | Other | 0.84 (0.65-1.08) | 0.18 |
| Parental education |  |  |  | Parental education |  |  |
| Lower (ref) | 1 |  |  | Lower (ref) | 1 |  |
| Intermediate | 0.98 (0.86-1.11) | 0.69 |  | Intermediate | 0.94 (0.82-1.07) | 0.32 |
| Higher | 0.90 (0.79-1.03) | 0.14 |  | Higher | 0.86 (0.75-0.99) | 0.031 |
| Education |  |  |  | Education |  |  |
| Less than high school (ref) | 1 |  |  | Less than high school (ref) | 1 |  |
| High school | 0.86 (0.74-1.00) | 0.057 |  | High school | 0.80 (0.68-0.94) | 0.007 |
| Some college | 0.82 (0.69-0.96) | 0.017 |  | Some college | 0.73 (0.61-0.87) | 0.001 |
| College and higher | 0.56 (0.47-1.03) | <0.001 |  | College and higher | 0.56 (0.47-0.67) | <0.001 |
| Cognition | 1.02 (1.01-1.03) | 0.001 |  | Cognition | 1.01 (1.00-1.03) | 0.032 |
| Life skills |  |  |  | Life skills |  |  |
| 0 (ref) | 1 |  |  | 0 (ref) | 1 |  |
| 1 | 0.94 (0.82-1.07) | 0.34 |  | 1 | 0.95 (0.83-1.09) | 0.45 |
| 2 | 0.95 (0.82-1.09) | 0.46 |  | 2 | 0.87 (0.75-0.99) | 0.047 |
| 3 | 0.86 (0.73-1.01) | 0.69 |  | 3 | 0.87 (0.74-1.02) | 0.091 |
| 4,5 | 0.70 (0.58-0.85) | <0.001 |  | 4,5 | 0.76 (0.63-0.91) | 0.003 |
| Linear trend across categories |  | <0.001 |  | Linear trend across categories |  | 0.002 |

**Table S8 Prospective associations of life skills in 2008/2010 with economic factors in 2014**

| **Wealth (% in top quintile, 2014)**  **(n = 6,871)** | | |  | **Income (% in top quintile, 2014)**  **(n = 6,871)** | | |
| --- | --- | --- | --- | --- | --- | --- |
| **Factor** | **Adjusted OR**  **(95% CI)** | **P** |  | **Factor** | **Adjusted OR**  **(95% CI)** | **P** |
| Age | 0.99 (0.98-0.99) | 0.017 |  | Age | 0.96 (0.95-0.97) | <0.001 |
| Sex | 0.89 (0.76-1.04) | 0.14 |  | Sex | 0.74 (0.63-0.87) | <0.001 |
| Ethnicity |  |  |  | Ethnicity |  |  |
| White (ref) | 1 |  |  | White (ref) | 1 |  |
| Black | 0.72 (0.51-1.03) | 0.073 |  | Black | 0.46 (0.34-0.64) | <0.001 |
| Other | 0.64 (0.40-1.03) | 0.068 |  | Other | 0.81 (0.53-1.23) | 0.31 |
| Parental education |  |  |  | Parental education |  |  |
| Lower (ref) | 1 |  |  | Lower (ref) | 1 |  |
| Intermediate | 1.02 (0.82-1.28) | 0.83 |  | Intermediate | 1.20 (0.94-1.55) | 0.15 |
| Higher | 1.11 (0.89-1.39) | 0.34 |  | Higher | 1.29 (1.01-1.63) | 0.041 |
| Education |  |  |  | Education |  |  |
| Less than high school (ref) | 1 |  |  | Less than high school (ref) | 1 |  |
| High school | 1.21 (0.87-1.67) | 1.21 |  | High school | 1.11 (0.83-1.68) | 0.64 |
| Some college | 1.47 (1.05-2.07) | 1.47 |  | Some college | 1.74 (1.14-2.65) | 0.011 |
| College and higher | 2.05 (1.46-2.88) | 2.05 |  | College and higher | 3.41 (2.24-5.20) | <0.001 |
| Cognition | 1.03 (1.01-1.06) | 0.004 |  | Cognition | 3.04 (2.78-3.32) | <0.001 |
| Wealth baseline | 10.21 (8.99-11.60) | <0.001 |  | Income baseline | 1.03 (1.01-1.06) | 0.009 |
| Life skills |  |  |  | Life skills |  |  |
| 0 (ref) | 1 |  |  | 0 (ref) | 1 |  |
| 1 | 1.12 (0.90-1.40) | 0.32 |  | 1 | 1.02 (0.81-1.29) | 0.85 |
| 2 | 1.18 (0.94-1.49) | 0.16 |  | 2 | 1.04 (0.82-1.32) | 0.73 |
| 3 | 1.13 (0.88-1.47) | 0.35 |  | 3 | 1.13 (0.87-1.47) | 0.37 |
| 4,5 | 1.31 (0.99-1.72) | 0.057 |  | 4,5 | 1.12 (0.84-1.48) | 0.45 |
| Linear trend across categories |  | 0.095 |  | Linear trend across categories |  | 0.38 |

**Table S9 Prospective associations of life skills in 2008/2010 with depression symptoms (2014) and anxiety (2012)**

| **Depressive symptoms (% above threshold)**  **(n = 6,579)** | | |  | **Anxiety (mean rating)**  **(n = 2,754)** | | |
| --- | --- | --- | --- | --- | --- | --- |
| **Factor** | **Adjusted OR**  **(95% CI)** | **P** |  | **Factor** | **Beta**  **(SE)** | **P** |
| Age | 1.00 (0.99-1.01) | 0.97 |  | Age | 0.038 (0.016) | 0.017 |
| Sex | 1.43 (1.20-1.71) | <0.001 |  | Sex | 0.043 (0.016) | 0.006 |
| Ethnicity |  |  |  | Ethnicity |  |  |
| White (ref) | 1 |  |  | black vs white | -0.007 (0.016) | 0.66 |
| Black | 1.20 (0.92-1.53) | 0.15 |  | other vs white | 0.028 (0.015) | 0.07 |
| Other | 1.68 (1.17-2.39) | 0.004 |  | Parental education |  |  |
| Parental education |  |  |  | intermediate v lower | -0.004 (0.019) | 0.84 |
| Lower (ref) | 1 |  |  | higher v lower | 0.029 (0.020) | 0.16 |
| Intermediate | 1.07 (0.86-1.31) | 0.56 |  | Education |  |  |
| Higher | 0.90 (0.72-1.13) | 0.38 |  | High school v less than HS | -0.021 (0.024) | 0.37 |
| Education |  |  |  | Some college v less than HS | -0.021 (0.023) | 0.37 |
| Less than high school (ref) | 1 |  |  | College and higher v less than HS | -0.020 (0.024) | 0.4 |
| High school | 0.72 (0.57-0.91) | 0.005 |  | Cognition | -0.056 (0.018) | 0.002 |
| Some college | 0.71 (0.54-0.93) | 0.011 |  | Anxiety baseline | 0.507 (0.017) | <0.001 |
| College and higher | 0.58 (0.43-0.79) | <0.001 |  | Life skills |  |  |
| Cognition | 0.96 (0.94-0.98) | <0.001 |  | 1 vs 0 | -0.006 (0.021) | 0.076 |
| Depression baseline | 8.42 (6.97-10.17) | <0.001 |  | 2 vs 0 | -0.096 (0.021) | <0.001 |
| Life skills |  |  |  | 3 vs 0 | -0.092 (0.020) | <0.001 |
| 0 (ref) | 1 |  |  | 4,5 vs 0 | -0.105 (0.019) | <0.001 |
| 1 | 0.69 (0.56-0.84) | <0.001 |  | Linear trend across categories |  | <0.001 |
| 2 | 0.45 (0.36-0.57) | <0.001 |  |  |  |  |
| 3 | 0.32 (0.23-0.44) | <0.001 |  |  |  |  |
| 4,5 | 0.25 (0.16-0.37) | <0.001 |  |  |  |  |
| Linear trend across categories |  | <0.001 |  |  |  |  |

**Table S10 Prospective associations of life skills in 2008/2010 with financial strain and chronic stress in 2014**

| **Financial strain (% above threshold)**  **(n = 3,518)** | | |  | **Chronic stress (mean rating)**  **(n = 3,076)** | | |
| --- | --- | --- | --- | --- | --- | --- |
| **Factor** | **Adjusted OR**  **(95% CI)** | **P** |  | **Factor** | **Beta**  **(SE)** | **P** |
| Age | 0.98 (0.97-0.99) | <0.001 |  | Age | -0.099 (0.017) | <0.001 |
| Sex | 1.07 (0.88-1.29) | 0.52 |  | Sex | -0.017 (0.015) | 0.25 |
| Ethnicity |  |  |  | Ethnicity |  |  |
| White (ref) | 1 |  |  | black vs white | 0.026 (0.016) | 0.1 |
| Black | 1.45 (1.11-1.90) | 0.006 |  | other vs white | -0.038 (0.016) | 0.015 |
| Other | 1.20 (0.79-1.83) | 0.40 |  | Parental education |  |  |
| Parental education |  |  |  | intermediate v lower | -0.014 (0.019) | 0.48 |
| Lower (ref) | 1 |  |  | higher v lower | -0.020 (0.021) | 0.35 |
| Intermediate | 1.07 (0.83-1.37) | 0.61 |  | Education |  |  |
| Higher | 0.86 (0.67-1.12) | 0.27 |  | High school v less than HS | -0.049 (0.025) | 0.053 |
| Education |  |  |  | Some college v less than HS | -0.020 (0.025) | 0.41 |
| Less than high school (ref) | 1 |  |  | College and higher v less than HS | -0.022 (0.026) | 0.4 |
| High school | 0.83 (0.62-1.12) | 0.23 |  | Cognition | -0.019 (0.017) | 0.29 |
| Some college | 0.94 (0.68-1.30) | 0.71 |  | Chronic stress baseline | 0.0498 (0.016) | <0.001 |
| College and higher | 0.63 (0.43-0.90) | 0.011 |  | Life skills |  |  |
| Cognition | 0.98 (0.95-1.00) | 0.069 |  | 1 vs 0 | -0.053 (0.019) | 0.005 |
| Financial strain baseline | 11.94 (9.84-14.49) | <0.001 |  | 2 vs 0 | 0.090 (0.019) | <0.001 |
| Life skills |  |  |  | 3 vs 0 | 0.093 (0.018) | <0.001 |
| 0 (ref) | 1 |  |  | 4,5 vs 0 | -0.077 (0.018) | <0.001 |
| 1 | 0.85 (0.67-1.09) | 0.20 |  | Linear trend across categories |  | <0.001 |
| 2 | 0.77 (0.59-0.99) | 0.049 |  |  |  |  |
| 3 | 0.51 (0.37-0.71) | <0.001 |  |  |  |  |
| 4,5 | 0.61 (0.41-0.91) | 0.015 |  |  |  |  |
| Linear trend across categories |  | <0.001 |  |  |  |  |

**Table S11 Prospective associations of life skills in 2008/2010 with loneliness and close relationships in 2014**

| **Loneliness (mean rating)**  **(n = 3,651)** | | |  | **Number of close relationships (mean rating)**  **(n = 3,566)** | | |
| --- | --- | --- | --- | --- | --- | --- |
| **Factor** | **Beta**  **(SE)** | **P** |  | **Factor** | **Beta**  **(SE)** | **P** |
| Age | -0.012 (0.015) | 0.43 |  | Age | 0.055 (0.015) | <0.001 |
| Sex | 0.041 (0.014) | 0.003 |  | Sex | 0.012 (0.014) | 0.41 |
| Ethnicity |  |  |  | Ethnicity |  |  |
| black vs white | -0.003 (0.015) | 0.85 |  | black vs white | 0.041 (0.015) | 0.007 |
| other vs white | -0.028 (0.014) | 0.050 |  | other vs white | 0.045 (0.015) | 0.003 |
| Parental education |  |  |  | Parental education |  |  |
| intermediate v lower | -0.001 (0.018) | 0.97 |  | intermediate v lower | -0.009 (0.018) | 0.62 |
| higher v lower | -0.024 (0.019) | 0.21 |  | higher v lower | 0.002 (0.020) | 0.91 |
| Education |  |  |  | Education |  |  |
| High school v less than HS | -0.043 (0.023) | 0.055 |  | High school v less than HS | -0.009 (0.024) | 0.72 |
| Some college v less than HS | -0.007 (0.022) | 0.76 |  | Some college v less than HS | -0.010 (0.023) | 0.66 |
| College and higher v less than HS | -0.005 (0.023) | 0.83 |  | College and higher v less than HS | -0.035 (0.024) | 0.15 |
| Cognition | -0.026 (0.016) | 0.10 |  | Cognition | 0.008 (0.017) | 0.62 |
| Loneliness baseline | 0.518 (0.015) | <0.001 |  | Close relationships baseline | 0.514 (0.014) | <0.001 |
| Life skills |  |  |  | Life skills |  |  |
| 1 vs 0 | -0.050 (0.018) | 0.004 |  | 1 vs 0 | 0.027 (0.018) | 0.13 |
| 2 vs 0 | -0.094 (0.018) | <0.001 |  | 2 vs 0 | 0.057 (0.018) | 0.001 |
| 3 vs 0 | -0.115 (0.017) | <0.001 |  | 3 vs 0 | 0.050 (0.017) | 0.003 |
| 4,5 vs 0 | -0.108 (0.016) | <0.001 |  | 4,5 vs 0 | 0.044 (0.016) | 0.007 |
| Linear trend across categories |  | <0.001 |  | Linear trend across categories |  | 0.001 |

**Table S12 Prospective associations of life skills in 2008/2010 and stopping volunteering in 2014**

| **Stopping volunteering (%)**  **(n = 1,338)** | | |  |
| --- | --- | --- | --- |
| **Factor** | **Adjusted OR**  **(95% CI)** | **P** |  |
| Age | 1 | 0.88 |  |
| Sex | 1.09 (0.85-1.40) | 0.49 |  |
| Ethnicity |  |  |  |
| White (ref) | 1 |  |  |
| Black | 0.52 (0.35-0.78) | 0.001 |  |
| Other | 1.11 (0.61-2.02) | 0.73 |  |
| Parental education |  |  |  |
| Lower (ref) | 1 |  |  |
| Intermediate | 1.26 (0.90-1.77) | 0.18 |  |
| Higher | 1.13 (0.81-1.59) | 0.48 |  |
| Education |  |  |  |
| Less than high school (ref) | 1 |  |  |
| High school | 0.83 (0.53-1.29) | 0.4 |  |
| Some college | 0.58 (0.36-0.93) | 0.58 |  |
| College and higher | 0.46 (0.30-0.78) | 0.48 |  |
| Cognition | 0.92 (0.89-0.95) | <0.001 |  |
| Life skills |  |  |  |
| 0 (ref) | 1 |  |  |
| 1 | 0.79 (0.56-1.10) | 0.16 |  |
| 2 | 0.78 (0.55-1.10) | 0.15 |  |
| 3 | 0.57 (0.38-0.84) | 0.005 |  |
| 4,5 | 0.54 (0.35-0.84) | 0.006 |  |
| Linear trend across categories |  | <0.001 |  |

**Table S13 Prospective associations of life skills in 2008/2010 and health in 2014**

| **Self-rated health (% deterioration)**  **(n = 5,417)** | | |  | **Chronic illness (mean number)**  **(n = 7,835)** | | |
| --- | --- | --- | --- | --- | --- | --- |
| **Factor** | **Adjusted OR**  **(95% CI)** | **P** |  | **Factor** | **Beta**  **(SE)** | **P** |
| Age | 1.02 (1.01-1.02) | 0.009 |  | Age | 0.016 (0.006) | 0.005 |
| Sex | 0.87 (0.75-1.02) | 0.32 |  | Sex | -0.012 (0.005) | 0.025 |
| Ethnicity |  |  |  | Ethnicity |  |  |
| White (ref) | 1 |  |  | black vs white | 0.006 (0.005) | 0.28 |
| Black | 1.00 (0.78-1.28) | 0.47 |  | other vs white | -0.003 (0.005) | 0.56 |
| Other | 1.26 (0.85-1.87) | 0.22 |  | Parental education |  |  |
| Parental education |  |  |  | intermediate v lower | -0.007 (0.006) | 0.26 |
| Lower (ref) | 1 |  |  | higher v lower | -0.012 (0.007) | 0.091 |
| Intermediate | 0.90 (0.74-1.09) | 0.15 |  | Education |  |  |
| Higher | 0.77 (0.63-0.95) | 0.021 |  | High school v less than HS | -0.003 (0.008) | 0.75 |
| Education |  |  |  | Some college v less than HS | 0.003 (0.008) | 0.74 |
| Less than high school (ref) | 1 |  |  | College and higher v less than HS | -0.014 (0.008) | 0.087 |
| High school | 0.71 (0.56-0.89) | <0.001 |  | Cognition | -0.008 (0.006) | 0.19 |
| Some college | 0.60 (0.47-0.78) | <0.001 |  | Chronic illness baseline | 0.880 (0.005) | <0.001 |
| College and higher | 0.49 (0.37-0.64) | <0.001 |  | Life skills |  |  |
| Cognition | 0.94 (0.92-0.96) | 0.001 |  | 1 vs 0 | -0.007 (0.007) | 0.27 |
| Life skills |  |  |  | 2 vs 0 | -0.015 (0.006) | 0.016 |
| 0 (ref) | 1 |  |  | 3 vs 0 | -0.006 (0.006) | 0.30 |
| 1 | 0.76 (0.62-0.93) | 0.064 |  | 4,5 vs 0 | -0.021 (0.006) | <0.001 |
| 2 | 0.63 (0.50-0.78) | 0.005 |  | Linear trend across categories |  | 0.001 |
| 3 | 0.47 (0.36-0.62) | <0.001 |  |  |  |  |
| 4,5 | 0.43 (0.32-0.58) | <0.001 |  |  |  |  |
| Linear trend across categories |  | <0.001 |  |  |  |  |
|  |  |  |  |  |  |  |

**Table S14 Prospective associations of life skills in 2008/2010 with impaired ADLs and gait speed in 2014**

| **Impaired ADLs (% incident impairment)**  **(n = 7,309)** | | |  | **Gait speed (mean)**  **(n = 2,045)** | | |
| --- | --- | --- | --- | --- | --- | --- |
| **Factor** | **Adjusted OR**  **(95% CI)** | **P** |  | **Factor** | **Beta**  **(SE)** | **P** |
| Age | 1.04 (1.03-1.05) | <0.001 |  | Age | -0.242(0.0182) | <0.001 |
| Sex | 1.33 (1.13-1.55) | <0.001 |  | Sex | -0.070 (0.018) | <0.001 |
| Ethnicity |  |  |  | Ethnicity |  |  |
| White (ref) | 1 |  |  | black vs white | -0.071 (0.018) | <0.001 |
| Black | 1.23 (0.97-1.55) | 0.093 |  | other vs white | 0.006 (0.017) | 0.074 |
| Other | 1.02 (9.67-1.56) | 0.92 |  | Parental education |  |  |
| Parental education |  |  |  | intermediate v lower | 0.009 (0.022) | 0.69 |
| Lower (ref) | 1 |  |  | higher v lower | 0.005 (0.023) | 0.83 |
| Intermediate | 0.86 (0.71-1.03) | 0.10 |  | Education |  |  |
| Higher | 0.83 (0.67-1.02) | 0.074 |  | High school v less than HS | 0.057 (0.029) | 0.049 |
| Education |  |  |  | Some college v less than HS | 0.100 (0.028) | <0.001 |
| Less than high school (ref) | 1 |  |  | College and higher v less than HS | 0.160 (0.030) | <0.001 |
| High school | 0.84 (0.68-1.05) | 0.12 |  | Cognition | 0.080 (0.020) | <0.001 |
| Some college | 0.76 (0.59-0.98) | 0.031 |  | Gait speed baseline | 0.419 (0.019) | <0.001 |
| College and higher | 0.71 (0.54-0.93) | 0.013 |  | Life skills |  |  |
| Cognition | 0.97 (0.95-0.99) | <0.001 |  | 1 vs 0 | 0.032 (0.022) | 0.14 |
| Life skills |  |  |  | 2 vs 0 | 0.060 (0.022) | 0.005 |
| 0 (ref) | 1 |  |  | 3 vs 0 | 0.060 (0.020) | 0.005 |
| 1 | 0.84 (0.69-1.03) | 0.097 |  | 4,5 vs 0 | 0.046 (0.020) | 0.019 |
| 2 | 0.80 (0.65-0.99) | 0.044 |  | Linear trend across categories |  | 0.006 |
| 3 | 0.67 (0.52-0.87) | 0.003 |  |  |  |  |
| 4,5 | 0.51 (0.38-0.70) | <0.001 |  |  |  |  |
| Linear trend across categories |  | <0.001 |  |  |  |  |
|  |  |  |  |  |  |  |

**Table S 15 Sensitivity analyses: life skill index excluding each component in turn**

|  | **Full index** | | **Excluding conscientiousness** | | **Excluding emotional stability** | | **Excluding persistence** | | **Excluding optimism** | | **Excluding control** | |
| --- | --- | --- | --- | --- | --- | --- | --- | --- | --- | --- | --- | --- |
|  | *OR, β^1^* | *p^2^* | *OR, β* | *p* | *OR, β* | *p* | *OR, β* | *p* | *OR, β* | *p* | *OR, β* | *p* |
| Wealth | 1.37 | 0.006 | 1.32 | 0.010 | 1.31 | 0.009 | 1.35 | 0.001 | 1.30 | 0.003 | 1.24 | 0.036 |
| Income | 1.45 | <0.001 | 1.38 | 0.001 | 1.43 | <0.001 | 1.27 | 0.002 | 1.35 | <0.001 | 1.35 | 0.001 |
| Depressive symptoms | 0.12 | <0.001 | 0.12 | <0.001 | 0.17 | <0.001 | 0.13 | <0.001 | 0.14 | <0.001 | 0.18 | <0.001 |
| Anxiety | -0.340 | <0.001 | -0.381 | <0.001 | -0.303 | <0.001 | -0.343 | <0.001 | -0.390 | <0.001 | -0.306 | <0.001 |
| Financial strain | 0.30 | <0.001 | 0.36 | <0.001 | 0.37 | <0.001 | 0.35 | <0.001 | 0.35 | <0.001 | 0.43 | <0.001 |
| Chronic stress | -0.206 | <0.001 | -0.229 | <0.001 | -0.193 | <0.001 | -0.245 | <0.001 | -0.230 | <0.001 | -0.176 | <0.001 |
| Social isolation | 0.61 | <0.001 | 0.622 | <0.001 | 0.64 | <0.001 | 0.71 | <0.001 | 0.67 | <0.001 | 0.69 | 0.001 |
| Loneliness | 0.12 | <0.001 | 0.14 | <0.001 | 0.17 | <0.001 | 0.15 | <0.001 | 0.16 | <0.001 | 0.20 | <0.001 |
| Close relationships | 0.095 | <0.001 | 0.120 | <0.001 | 0.109 | <0.001 | 0.110 | <0.001 | 0.109 | <0.001 | 0.081 | <0.001 |
| Volunteering | 1.61 | <0.001 | 1.57 | <0.001 | 1.52 | <0.001 | 1.56 | <0.001 | 1.53 | <0.001 | 1.56 | <0.001 |
| Self-rated health | 0.13 | <0.001 | 0.32 | <0.001 | 0.35 | <0.001 | 0.29 | <0.001 | 0.33 | <0.001 | 0.37 | <0.001 |
| Chronic disease | 0.53 | <0.001 | 0.61 | <0.001 | 0.58 | <0.001 | 0.54 | <0.001 | 0.59 | <0.001 | 0.60 | <0.001 |
| Impaired ADLs | 0.23 | <0.001 | 0.40 | <0.001 | 0.40 | <0.001 | 0.33 | <0.001 | 0.37 | <0.001 | 0.43 | <0.001 |
| Gait speed | 0.086 | <0.001 | 0.079 | <0.001 | 0.079 | <0.001 | 0.083 | <0.001 | 0.084 | <0.001 | 0.061 | <0.001 |
| Waist circumference | 0.67 | <0.001 | 0.81 | 0.013 | 0.75 | <0.001 | 0.82 | 0.008 | 0.82 | 0.006 | 0.81 | 0.004 |
| Obesity | 0.74 | <0.001 | 0.82 | 0.079 | 0.75 | <0.001 | 0.76 | 0.001 | 0.79 | 0.003 | 0.72 | <0.001 |
|  |  |  |  |  |  |  |  |  |  |  |  |  |
| **Longitudinal results** |  |  |  |  |  |  |  |  |  |  |  |  |
| Depressive symptoms | 0.25 | <0.001 | 0.25 | <0.001 | 0.40 | <0.001 | 0.23 | <0.001 | 0.28 | <0.001 | 0.29 | <0.001 |
| Anxiety | -0.105 | <0.001 | -0.136 | <0.001 | -0.100 | <0.001 | -0.132 | <0.001 | -0.116 | <0.001 | -.080 | <0.001 |
| Financial strain | 0.61 | 0.001 | 0.53 | <0.001 | 0.60 | 0.001 | 0.55 | <0.001 | 0.58 | <0.001 | 0.68 | 0.012 |
| Chronic stress | -0.077 | <0.001 | -0.086 | <0.001 | -0.088 | <0.001 | -0.106 | <0.001 | -0.096 | <0.001 | -0.057 | <0.001 |
| Loneliness | -0.108 | <0.001 | -0.118 | <0.001 | -0.107 | <0.001 | -0.144 | <0.001 | -0.125 | <0.001 | -0.095 | <0.001 |
| Close relationships | 0.044 | 0.001 | 0.049 | <0.001 | 0.048 | 0.001 | 0.055 | 0.001 | 0.046 | 0.001 | 0.046 | 0.003 |
| Volunteering | 0.54 | 0.001 | 0.58 | 0.001 | 0.59 | 0.001 | 0.51 | 0.001 | 0.60 | 0.003 | 0.59 | <0.001 |
| Self-rated health | 0.48 | <0.001 | 0.48 | <0.001 | 0.49 | <0.001 | 0.44 | <0.001 | 0.46 | <0.001 | 0.48 | <0.001 |
| Chronic disease | -0.021 | 0.001 | -0.019 | 0.003 | -0.014 | 0.011 | -0.019 | 0.001 | -0.019 | 0.002 | -0.018 | 0.006 |
| Impaired ADLs | 0.48 | <0.001 | 0.55 | <0.001 | 0.67 | <0.001 | 0.59 | <0.001 | 0.55 | <0.001 | 0.59 | <0.001 |
| Gait speed | 0.034 | 0.006 | 0.058 | 0.001 | 0.047 | 0.014 | 0.057 | 0.003 | 0.067 | 0.001 | 0.038 | 0.066 |

^1^ Adjusted odds ratio (OR) for the highest life skill category, or standardized regression coefficient *β* for continuously distributed outcomes. Results for continuously distributed variables are shown with 3 decimal points, and OR with 2 points. All analyses are adjusted for age, sex, ethnicity, parental education, own education, and cognitive function.

^2^ *P* is for linear gradients across life skill categories.

**Table S16 Sensitivity analyses**

|  | **Full index** | | **Wealth and self-rated health covariates** | | **Continuous life skills**  **index** | | **White participants** | |
| --- | --- | --- | --- | --- | --- | --- | --- | --- |
|  | *OR, β^1^* | *p^2^* | *OR, β* | *p* | *OR, β* | *p* | *OR, β* | *p* |
| Wealth | 1.37 | 0.006 |  |  | 1.25 | <0.001 | 1.40 | 0.002 |
| Income | 1.45 | <0.001 | 1.23 | 0.027 | 1.37 | <0.001 | 1.37 | 0.002 |
| Depressive symptoms | 0.12 | <0.001 | 0.16 | <0.001 | 0.44 | <0.001 | 0.01 | <0.001 |
| Anxiety | -0.340 | <0.001 | -0.309 | <0.001 | -0.184 | <0.001 | -0.346 | <0.001 |
| Financial strain | 0.30 | <0.001 | 0.33 | <0.001 | 0.64 | <0.001 | 0.27 | <0.001 |
| Chronic stress | -0.206 | <0.001 | -0.183 | <0.001 | -0.114 | <0.001 | -0.218 | <0.001 |
| Social isolation | 0.61 | <0.001 | 0.65 | <0.001 | 0.64 | <0.001 | 0.62 | <0.001 |
| Loneliness | 0.12 | <0.001 | 0.13 | <0.001 | 0.43 | <0.001 | 0.10 | <0.001 |
| Close relationships | 0.095 | <0.001 | 0.088 | <0.001 | 0.136 | <0.001 | 0.093 | <0.001 |
| Volunteering | 1.61 | <0.001 | 1.50 | <0.001 | 1.52 | <0.001 | 1.60 | <0.001 |
| Self-rated health | 0.13 | <0.001 | - |  | 0.52 | <0.001 | 0.23 | <0.001 |
| Chronic disease | 0.53 | <0.001 | 0.63 | <0.001 | 0.79 | <0.001 | 0.55 | <0.001 |
| Impaired ADLs | 0.23 | <0.001 | 0.43 | <0.001 | 0.58 | <0.001 | 0.30 | <0.001 |
| Gait speed | 0.086 | <0.001 | 0.059 | <0.001 | 0.101 | <0.001 | 0.074 | <0.001 |
| Waist circumference | 0.67 | <0.001 | 0.81 | 0.035 | 0.80 | <0.001 | 0.74 | 0.002 |
| Obesity | 0.74 | <0.001 | 0.79 | 0.028 | 0.82 | <0.001 | 0.67 | <0.001 |
|  |  |  |  |  |  |  |  |  |
| **Longitudinal results** |  |  |  |  |  |  |  |  |
| Depressive symptoms | 0.25 | <0.001 | 0.29 | <0.001 | 0.60 | <0.001 | 0.18 | <0.001 |
| Anxiety | -0.105 | <0.001 | -0.098 | <0.001 | -0.050 | 0.002 | -0.104 | <0.001 |
| Financial strain | 0.61 | 0.001 | 0.64 | 0.002 | 0.72 | <0.001 | 0.56 | <0.001 |
| Chronic stress | -0.077 | <0.001 | -.071 | <0.001 | -0.070 | <0.001 | -0.086 | <0.001 |
| Loneliness | -0.108 | <0.001 | -0.101 | <0.001 | -0.068 | <0.001 | -0.110 | <0.001 |
| Close relationships | 0.044 | 0.001 | 0.038 | 0.009 | 0.059 | <0.001 | 0.041 | 0.013 |
| Volunteering | 0.54 | 0.001 | 0.59 | 0.005 | 0.81 | 0.12 | 0.48 | 0.001 |
| Self-rated health | 0.48 | <0.001 | - |  | 0.62 | <0.001 | 0.62 | 0.007 |
| Chronic disease | -0.021 | 0.001 | -0.019 | 0.006 | -0.009 | 0.093 | -0.027 | <0.001 |
| Impaired ADLs | 0.48 | <0.001 | 0.55 | <0.001 | 0.79 | 0.003 | 0.52 | <0.001 |
| Gait speed | 0.034 | 0.006 | 0.034 | 0.038 | 0.023 | 0.19 | 0.058 | 0.001 |

^1^ Adjusted odds ratio (OR) for the highest life skill category, or standardized regression coefficient *β* for continuously distributed outcomes. Results for continuously distributed variables are shown with 3 decimal points, and OR with 2 points. All analyses are adjusted for age, sex, ethnicity (except for analyses of White participants), parental education, own education, and cognitive function.

^2^ *P* is for linear gradients across life skill categories.
